# 

**Published:** 1829-05

**Authors:** 


### MONTHLY LIST OF MEDICAL BOOKS.

[*Medical Works cannot be entered on this List except a copy be sent for the purpose; the titles of Books having frequently been transmitted to us, as published, which have not appeared for weeks, or even months, after.*]

An Essay on the Use of the Nitrate of Silver in the Cure of Inflammation, Wounds, and Ulcers. By John Higginbottom, Nottingham, Surgeon. Second Edition, much improved and enlarged.—8vo. pp. 204. London, 1829.

On the Varieties of Deafness and Diseases of the Ear, with proposed Methods of relieving them. By Wm. Wright, Esq. Surgeon Aurist to her late Majesty Queen Charlotte, &c.—8vo. pp. 295. London, 1829.

Annali Universali di Medicina.

A Pocket Compendium of Anatomy, containing a correct and accurate Description of the Human Body. By E. W. TUSON, Lecturer on Anatomy, &c.—12mo. pp. 289. London, 1828.

Pathological and Practical Researches on Diseases of the Stomach, the Intestinal Canal, the Liver, and other Viscera of the Abdomen. By JOHN ABERCROMBIE, M.D. &c.—8vo. pp. 396. London, 1828.

A Clinical Lecture delivered to the Students of Surgery in the Royal Infirmary of Edinburgh, at the conclusion of the Winter Course for 1828-1829. By GEORGE BALLINGALL, M.D. F.R.S.E. &c.

*** We shall shortly present our readers with the whole of this very interesting practical lecture.

An Account of the Morbid Appearances exhibited on Dissection in Disorders of the Trachea, Lungs, and Heart; with Pathological Observations, to which a Comparison of the Symptoms with the Morbid Change has given rise. By THOMAS MILLS, M.D. &c.—8vo. pp. 302. Dublin. London, 1829.

Further Observations on the Use of the Lancetted Stilettes, in the Cure of Permanent Strictures of the Urethra. With additional Cases. By RICHARD ANTHONY STAFFORD, Member of the Royal College of Surgeons, and lately House-Surgeon to St. Bartholomew's Hospital.—8vo. Longman, 1829.

### NOTICES.

*Communications have been received from* Dr. J. WILSON, R.N. *(presented by* Dr. BURNETT ;) *and* Dr. GORDON.

